# Assessment of Affect Lability: Psychometric Properties of the ALS-18

**DOI:** 10.3389/fpsyg.2018.00427

**Published:** 2018-03-29

**Authors:** Anna Contardi, Claudio Imperatori, Italia Amati, Michela Balsamo, Marco Innamorati

**Affiliations:** ^1^Department of Human Sciences, European University of Rome, Rome, Italy; ^2^Dipartimento di Tecnologie, Comunicazione e Società, Università degli Studi Guglielmo Marconi, Rome, Italy; ^3^Dipartimento di Scienze Psicologiche, della Salute e del Territorio, Università degli Studi “G. d’Annunzio” Chieti-Pescara, Chieti, Italy

**Keywords:** affect lability, emotion dysregulation, Affective Lability Scales (ALS-18), psychometric properties of ALS-18, validity and reliability of ALS-18

## Abstract

Affect lability, an important aspect of emotion dysregulation, characterizes several psychiatric conditions. The short Affective Lability Scales (ALS-18) measures three aspects of changeability between euthymia and affect states (Anxiety/Depression, AD; Depression/Elation, DE; and Anger, Ang). The aim of our study was to investigate the psychometric characteristics of an Italian version of the ALS-18 in a sample of adults recruited from the general population. The sample was composed of 494 adults (343 women and 151 men) aged 18 and higher (mean age = 31.73 years, *SD* = 12.6). All participants were administered a checklist assessing socio-demographic variables, the ALS-18 and measures of depression and difficulties in emotion regulation. Confirmatory factor analyses indicated adequate fit of the three-factor model (RMSEA = 0.061, 95% CI = 0.054/0.069; CFI = 0.99; SRMR = 0.055), and the presence of a higher-order general factor. Internal consistency was satisfactory for all the lower-order dimensions and the general factor (ordinal α > 0.70). The ALS-18 was significantly associated with concurrent measures of depression and difficulties in emotion regulation. These findings indicate that the ALS-18 is a valid and reliable instrument for measuring affect lability, although discriminant validity of subdimensions scores could be problematic.

## Introduction

Past studies suggested that emotion dysregulation could be associated with the development and maintenance of various psychiatric disorders and maladaptive behaviors (Amstadter, 2008; Aldao et al., 2010; Svaldi et al., 2012; American Psychiatric Association, 2013; Contardi et al., 2013). An important aspect of emotion dysregulation is affect lability intended as abnormally frequent, intense, and wide ranging changes in affective states (Thompson et al., 2011). Affect lability is present in several psychiatric conditions and is characteristic of the bipolar disorder and the borderline personality disorder (Henry et al., 2008; Aminoff et al., 2012; Reich et al., 2012). For example, affect lability has recently been found to mediate the relationship between childhood trauma and suicide attempts in bipolar patients (Aas et al., 2017).

The construct of affect lability has strong relationships with other personological constructs such as neuroticism (McCrae and Costa, 1987) and cyclothymia (Akiskal, 2001). Neuroticism (also known as emotional stability-instability or negative emotionality) is part of major models of normal personality structure (i.e., Eysenck’s Three Factor model, and the Big Five model) and it is an ubiquitous element of many personality measures (McCrae and Costa, 1987; Zuckerman et al., 1993). Negative emotionality is a central component in neuroticism, along with cognitive and behavioral facets (McCrae and Costa, 1987). For example, in the NEO Personality Inventory (NEO-PI-3) neuroticism is composed of six facets (i.e., Anxiety, Angry Hostility, Depression, Self-Consciousness, Impulsiveness, and Vulnerability) (McCrae et al., 2005). Questionnaires assessing neuroticism generally measure the frequency of negative emotional states and how easily they are experienced by the individual (e.g., “Get stressed out easily.”, “Often feel blue.”, “Lose my temper.”) (Maples et al., 2014). Conversely, measures of affect lability assess how frequently emotionality change between two specific polarized emotions (e.g., “At times I feel just as realized as everyone else and then within minutes I become so nervous that I feel light-headed and dizzy.”, “I switch back and forth between being extremely energetic and having so little energy that it’s a huge effort just to get where I’m going.”). Thus, although affect lability could be considered a facet of neuroticism, it has a close relationship with the psychiatric construct of cyclothymia. Kraepelin described the cyclothymic disposition as one of the constitutional substrates of the manic-depressive illness (Akiskal, 2001). According to Akiskal (2001) cyclothymic individuals report short cycles of mood swings, characterized mainly by depression and hypomania but also by labile-angry-irritable moods. Mood swings are essentially biphasic, with lethargy alternating with eutonia, or unexplained tearfulness alternating with excessive punning and jocularity (Akiskal and Mallya, 1987).

To measure affect lability, Harvey et al. (1989) developed the Affective Lability Scales (ALS), a 58-item questionnaire measuring changeability among euthymia and four affect states (i.e., depression, elation, anger, and anxiety). The four studies presented in their research indicated satisfactory reliability (internal consistency and stability), discriminant validity with a measure of affect intensity, and concurrent validity with measures of depression (Harvey et al., 1989). Nevertheless, Oliver and Simons (2004) considered the ALS too lengthy and developed a 18-item short form (ALS-18) consisting of at least two items from each dimensions of the ALS. In a non-clinical sample of university students a confirmatory factor analysis supported the adequacy of both a three-factor structure (Anxiety/Depression, AD; Depression/Elation, DE, Anger, Ang) (Bentler–Bonnett Non-normed Fit Index [NNFI] = 0.90; Comparative Fit Index [CFI] = 0.92; Root Mean Square Error of Approximation [RMSEA] = 0.06), and a six-factor model reflecting the structure of the original 58-item version (NNFI = 0.94; CFI = 0.96; RMSEA = 0.05) (Oliver and Simons, 2004). However, the six-factor model included two dimensions composed of only 2 items (Elation and Hypomania), and internal consistency was found to be lower than for the three-factor model. Further studies investigated successfully the adequacy of the three-factor model in different clinical populations (e.g., personality disorders, bipolar disorder patients and relatives, and ADHD) (Look et al., 2010; Aas et al., 2015; Weibel et al., 2017). For example, Look et al. (2010) investigated factor structure and psychometric properties of the ALS-18 in patients with personality disorders and individuals without any psychiatric conditions, and reported satisfactory reliability and good discriminant validity (i.e., people with DSM-IV Cluster B personality disorders reported higher scores than individuals with Cluster A and Cluster C disorders, and people without any psychiatric condition) (Look et al., 2010). Discriminant validity was also supported when differentiating ADHD patients from healthy controls (Weibel et al., 2017), or bipolar patients from relatives and healthy controls (Aas et al., 2015).

**FIGURE 1:**
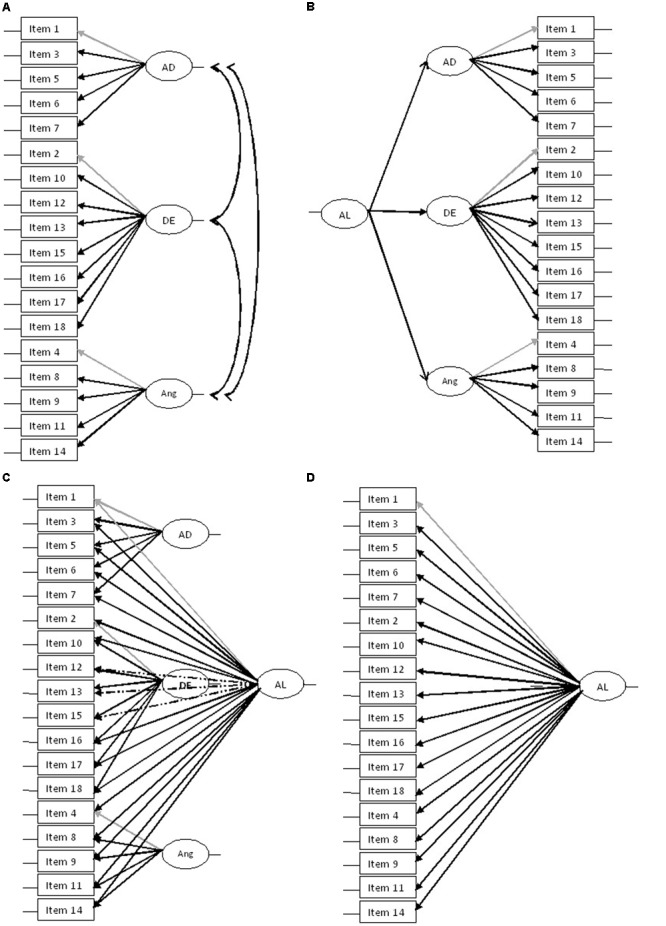
Structural models **(A)** Three factor model, **(B)** Hierarchical three factor model, **(C)** Bifactor model, and **(D)** One-factor model.

Based on the results presented above, and given that the psychometric characteristics of an Italian version of the ALS-18 (as well as the original 54-item version) have not already been investigated, the aim of our study was to investigate factor structure, validity and reliability of the Italian version of the ALS-18 in a non-clinical sample of adults from the general population, as a first step for a cross-cultural validation of the questionnaire. In line with previous studies (Look et al., 2010; Aas et al., 2015; Weibel et al., 2017), we tested the fit of a three-factor model and its superiority over a one-factor model. Considering that previous studies indicated that dimensions of the ALS-18 could be strongly correlated (*r* ≥ 0.64) (Amstadter, 2008; Look et al., 2010), we also tested whether a hierarchical factor model, with three specific factors (AD, DE, and Ang) loading on a higher-order general factor, or a bi-factor model, with each items loading upon both a group factor (AD, DE, and Ang) and a general factor (AL) could represent well the factor structure of the ALS-18 (**Figure 1**).

## Materials and Methods

### Participants and Procedure

The sample was composed of 494 adults (343 women and 151 men). Mean age of the participants was 31.73 years (*SD* = 12.61). Inclusion criteria were ages of 18 and higher. Exclusion criteria were the presence of any condition affecting the ability to take the assessment, including illiteracy or denial of informed consent. The sample was recruited through advertisements (flyers, newspaper and online ads) posted for established community groups, and directly from university communities (*n* = 194) of the authors of the present research. Individuals were approached by psychologists who informed them about the aim of the study and explained how to fill-in the questionnaire. They participated in the study voluntarily and received no payment. Each participant provided written, informed consent prior to data collection. The study protocol received ethics approval from the local research ethics review board. Sociodemographic characteristics of the sample are reported in **Table 1**.

**Table 1:** Characteristics of the sample (*n* = 494).

Variables	Percentages (Mean ±*SD*)
Sex	
Men	30.6%
Women	69.4%
Age – *M*(*SD*)	(31.73 ± 12.61)
Marital status	
Not-married (including widowed and divorced)	79.7%
Married	20.3%
School attainment (number of years)	
≤8	7.1%
≤13	53.8%
≥16	39.1%
Job status	
Student	43.6%
Employed	47.2%
Unemployed	5.1%
Retired	4.1%

*SD, standard deviation.*

### Measures

At entry into the study, all participants were administered a checklist assessing socio-demographic variables (sex, age, marital status, job, and school attainment), and the Italian version of the ALS-18, the Teate Depression Inventory (TDI; Balsamo et al., 2014; Balsamo and Saggino, 2014), and the Difficulties in Emotion Regulation Scale (DERS) (Gratz and Roemer, 2004).

The original version of the ALS-18 is an 18-item self-report measure used to assess the affect lability. Items are rated on a 4-point Likert type scale (from 0 = very uncharacteristic of me to 3 = very characteristic of me). In the present study, we used an Italian adaptation of this scale. Two bilingual researchers adapted the present version of the questionnaire from the original English version using the back-translation procedure.

The TDI is a 21-item self-report instrument designed to assess major depressive disorder as specified by the latest editions of the Diagnostic and Statistical Manual of Mental Disorders (DSM; American Psychiatric Association, 2000, 2013), in order to overcome psychometric weaknesses of existing measures of depression (Balsamo and Saggino, 2007). Each item is rated on a five-point Likert-type scale, ranging from 0 (always) to 4 (never). The TDI demonstrated good psychometric properties (Balsamo et al., 2013, 2014, 2015a,b,c; Innamorati et al., 2013; Saggino et al., 2017). In the present sample, Cronbach’s α was 0.93.

The DERS is a 36-item multidimensional self-report measure assessing the individual’s characteristic patterns of emotion regulation. Items are rated on a 5-point Likert-type scale (from 1 = *almost never* to 5 = *almost always*) indicating the degree to which each statement describes the respondent’s behavior. It contains the following six subscales: (1) *Non-acceptance* of emotional responses; (2) Difficulties engaging in goal-directed behavior when experiencing negative emotions (*Goals*); (3) Impulse control difficulties when experiencing negative emotions (*Impulse*); (4) *L*ack of emotional *awareness*; (5) *L*imited access to emotion regulation *strategies* that are perceived as effective; and (6) *L*ack of emotional *clarity*. In the current sample internal consistency ranged between 0.76 for Awareness and 0.88 for Acceptance.

### Statistical Analysis

All the analyses were performed with the Statistical Package for the Social Sciences (SPSS) 19.0 for Windows, and Lisrel 8.80 (Jöreskog and Sörbom, 2006).

Confirmatory factor analysis was performed using a Robust Diagonally Weighted Least Squares estimator (DWLSE) with a polychoric correlation matrix. Model fit was assessed using the following indices: (1) the Root Mean Square Error of Approximation (RMSEA), with values between 0.05 and 0.08 indicative of adequacy of the model, and values below 0.05 indicating evidence of good fit (Browne and Cudek, 1993; Hu and Bentler, 1999); (2) the Comparative Fit Index (CFI), with values greater than 0.95/0.96 indicating good fit of the model; (3) the Standardized Root Mean Square Residual (SRMR), with values of less than 0.08 indicating good fit (Hu and Bentler, 1999); and (4) the Satorra-Bentler scaled chi-square (*χ^2^*) test and the normed *χ^2^* (*χ^2^*/degrees of freedom). *P*-values for the *χ^2^* test greater than 0.05 and a normed *χ^2^* less than 3 (Schreiber et al., 2006) indicate that the model is an adequate fit to the data, although the *χ^2^* test over-reject true models for large samples. The Expected Cross-validation Index (ECVI) was used to compare competing models (Browne and Cudeck, 1989).

Although the aim of the present study was to compare the four competing factor models and select the one with the best fit, the proposed three-factor model and the hierarchical three-factor model are equivalent (i.e., each factor directly or indirectly is related to all the other latent variables) and yield the same fit to the data (MacCallum et al., 1993; Leone, 2009). Thus, the comparison of fit indices is inconclusive in demonstrating which of the two models is better.

As measures of reliability, we reported ordinal Cronbach’s alpha (*α*) (Zumbo et al., 2007). Associations with sociodemographic variables and other measures were evaluated by means of a series of *t*-tests and Pearson’s *r* indices of correlations.

## Results

### Confirmatory Factor Analysis

The bi-factor model did not converge, and the statistical software issued a warning message indicating that Phi (i.e., the variance/covariance matrix between latent variables) was not positive definite. The other competing models all had significant χ^2^ (*p* < 0.001), indicating potential misfit of the models (see **Table 2**). On the contrary other fit indices indicated the adequacy of both the one-factor model (RMSEA = 0.072, 95% CI = 0.064/0.079; CFI = 0.98; SRMR = 0.066), and the three factor model (RMSEA = 0.061, 95% CI = 0.054/0.069; CFI = 0.99; SRMR = 0.055). Nevertheless, the ECVI suggested the superiority of the three-factor model (0.93 vs. 1.41).

**Table 2:** Fit indices for the competing factor models.

Model	χ^2^ (df)	RMSEA (90%CI)	CFI	SRMR	ECVI (90% CI)
Original three-factor model	369.02^∗^ (132)	0.061 (0.054/0.069)	0.99	0.054	0.93 (0.81/1.05)
Three-factor hierarchical model	369.02^∗^ (132)	0.061 (0.054/0.069)	0.99	0.054	0.93 (0.81/1.05)
One-factor model	610.30^∗^ (135)	0.085 (0.079/0.092)	0.98	0.066	1.41 (1.26/1.58)

*^∗^*p* < 0.001.*

The latent dimensions of the three-factor model were highly correlated (*r* between 0.83 for AD/DE and 0.93 for AD/Ang), and when modeling a hierarchical factor model, factor loadings on the higher-order general factor were all significant (0.93 for AD, 0.89 for DE and 0.99 for Ang). Each item of the ALS-18 loaded significantly on its hypothesized dimension (**Table 3**).

**Table 3:** Standardized factor loadings for the ALS-18 items (*n* = 494).

Items	AD	Items	DE	Items	Ang
ALS1	0.83	ALS2	0.66	ALS4	0.80
ALS3	0.86	ALS10	0.77	ALS8	0.85
ALS5	0.84	ALS12	0.86	ALS9	0.87
ALS6	0.72	ALS13	0.62	ALS11	0.69
ALS7	0.92	ALS15	0.72	ALS14	0.79
		ALS16	0.85		
		ALS17	0.62		
		ALS18	0.61		
Ordinal alpha	0.91		0.89		0.89

*AD, Anxiety/Depression; DE, Depression/Elation; Ang, Anger.*

### Psychometric Properties of the ALS-18

Considering that fit indices suggested that the three-factor model could represent better the latent structure of the ALS-18, the following analyses will be based on this factor model. Internal consistency of the ALS-18 was satisfactory for all the lower-order dimensions (**Table 4**), and for the general factor (ordinal alpha = 0.95). Scores on the ALS-18 were not associated with sex (*p* > 0.05 for *t*-tests), or with age (*r* between -0.09 for DE and -0.14 for AD). The ALS-18 dimensions and the general factor were all significantly associated with concurrent measures of depression and difficulties in emotion regulation (**Table 4**). Correlations with the TDI were all significant and moderate (*r* ≥ 0.4), ranging from 0.47 for Ang to 0.59 for AD.

**Table 4:** Correlations between measures and descriptive statistics (*n* = 494).

	AD	DE	Ang	AL	TDI	Non-acceptance	Goals	IMPULSE	Awareness	Strategies	Clarity
*M* ±*SD*	0.68 ± 0.62	0.63 ± 0.50	0.54 ± 0.58	0.62 ± 0.51	26.44 ± 14.02	13.02 ± 5.87	13.54 ± 3.93	12.89 ± 5.23	14.67 ± 4.73	16.69 ± 6.67	11.14 ± 4.33
AD	–				0.59^∗∗^	0.54^∗∗^	0.43^∗∗^	0.51^∗∗^	0.15^∗∗^	0.58^∗∗^	0.48^∗∗^
DE	0.70^∗∗^	–			0.52^∗∗^	0.44^∗∗^	0.38^∗∗^	0.43^∗∗^	0.10^∗^	0.46^∗∗^	0.40^∗∗^
Ang	0.78^∗∗^	0.74^∗∗^	–		0.47^∗∗^	0.44^∗∗^	0.34^∗∗^	0.49^∗∗^	0.16^∗∗^	0.47^∗∗^	0.36^∗∗^
AL	0.90^∗∗^	0.92^∗∗^	0.91^∗∗^	–	0.58^∗∗^	0.52^∗∗^	0.42^∗∗^	0.52^∗∗^	0.15^∗∗^	0.55^∗∗^	0.46^∗∗^

*^∗∗^Significant for *p* < 0.01; Significant for *p* < 0.05. AD, Anxiety/Depression; DE, Depression/Elation; Ang, Anger; AL, Affect Lability; TDI, Teate Depression Inventory.*

## Discussion

In our sample, the three-factor model fitted the data well. This is in line with previous studies which evaluated the structure of other versions of the ALS-18 (Look et al., 2010; Aas et al., 2015; Weibel et al., 2017). Our results could also support the presence of a higher-order general factor suggesting the possibility to compute a total score as generally reported in the literature (Look et al., 2010; Aas et al., 2015; Weibel et al., 2017). Nevertheless, when comparing the three-factor and the hierarchical models the design of our study was not conclusive in demonstrating the superiority of one model over the other (MacCallum et al., 1993; Leone, 2009). Furthermore, the bi-factor model was empirical underidentified denoting that our study did not permit a test of the hypothesized bi-factor model (Green and Yang, 2017). However, as far as we know, this was the first temptative study which assessed directly the fit of a hierarchical or bifactor model for the ALS-18.

Inter-correlations among the three dimensions of the ALS-18 were high (*r* ≥ 0.83), and despite also other studies reported strong intercorrelations among latent factors (Amstadter, 2008; Look et al., 2010), our figures are higher than those reported in those studies. These data could indicate non-satisfactory discriminant validity of subdimensions scores (Look et al., 2010). The three dimensions and the general factor all had adequate internal consistency.

ALS-18 subdimensions and total score were not associated with sex or age. In the past only Harvey et al. (1989) have investigated this topic for the 54-item ALS and reported sex differences for the depression scale only, suggesting a possible tendency for men to experience depression as a more transient and changeable phenomenon than do women. Conversely, the ALS-18 subdimensions and the general factor were all significantly associated with concurrent measures of depression and difficulties in emotion regulation. Our results are partially discordant from findings of previous studies. For example, Oliver and Simons (2004), in a non-clinical sample of university students, reported significant but negative correlations between the ALS-18 and the Center for Epidemiologic Studies-Depression Scale (*r* between -0.33 for Ang and -0.47 for AD). Conversely in our sample, the correlations were all significant and positive. Unfortunately, Oliver and Simons (2004) did not comment this result in their article. Nevertheless, Weibel et al. (2017), who administered a short version of the Beck Depression Inventory (BDI), reported an *r* of 0.34 (*p* < 0.05) between the ALS-18 total score and the BDI. The positive association between depression and affect lability possibly indicates that people with higher lability could experience phases of depression, despite the two constructs are only moderately correlated and both depression and affect lability should be assessed independently (Weibel et al., 2017). The results could also be seen as supportive of the concept of “soft bipolars spectrum” (Akiskal and Mallya, 1987). In fact, several patients who receive a diagnosis of major depression (MDD) have subthreshold symptoms of bipolarity, which includes biphasic mood swings and cyclothymic traits. These patients differ from pure MDD patients and patients with bipolar disorder for their temperamental profile and for clinical variables (Innamorati et al., 2015). Innamorati et al. (2015), investigating the role of cyclothymic temperament in characterizing mood disorder patients, evidenced that around 39% of inpatients with unipolar depression could be included in the soft bipolar spectrum according to their affective temperament. These patients seem to differ from patients with pure major mood disorders for levels of hopelessness and suicide risk.

Correlations with the DERS were also generally moderate (*r* ≥ 0.4) with the exclusion of the dimension Awareness of the DERS whose correlations with the ALS-18 were weak (*r* between 0.10 and 0.16). This means that also the relationship between affect lability and difficulties in emotion regulation could be complex with a partial independence of these two constructs.

Our results must be considered in light of some issues referred to the design of the study. First, in our sample there was a disproportion of female participants compared to males probably associated with the recruitment of university students from the authors’ university communities. This bias also prevented us from assessing structural invariance of the questionnaire between sex. Second, our results are based on a general community sample of adults, composed mostly of young adults, which limits the generalizability of these findings to clinical conditions or older adults. Third, we administered only self-report measures potentially affected by social desirability. In conclusion, this may considered only a first necessary step in the process of the cross-cultural validation of the ALS-18.

## Conclusion

Our results indicate that the Italian version of the ALS-18 can produce valid and reliable assessments of affect lability. Additional studies are needed from clinical samples or samples of older adults with further psychometric assessments of reliability and measurement errors to draw clear recommendations for clinical practice use and research.

## Ethics Statement

All procedures performed in studies involving human participants were in accordance with the ethical standards of the institutional and/or national research committee and with the 1964 Helsinki declaration and its later amendments or comparable ethical standards.

## Author Contributions

AC was involved in the study conception and design, acquisition, analysis and interpretation of data, and drafting of the manuscript. CI was involved in the study conception and design, interpretation of data, and the critical revision of the manuscript, provided final approval of the version to be published. IA was involved in the acquisition of data, interpretation of data, and the critical revision of the manuscript. MB was involved in the study conception and design and the analysis and interpretation of data. MI was involved in the study conception and design, acquisition of data, analysis and interpretation of data, and drafting of the manuscript, and provided final approval of the version to be published.

## Conflict of Interest Statement

The authors declare that the research was conducted in the absence of any commercial or financial relationships that could be construed as a potential conflict of interest. The reviewer EP and handling Editor declared their shared affiliation.
